# Immunobiotic potential of fermented snail meat hydrolysate in local chicken with low protein content

**DOI:** 10.5455/javar.2024.k749

**Published:** 2024-03-28

**Authors:** Ujang Suryadi, Rosa Tri Hertamawati, Shokhirul Imam

**Affiliations:** Department of Animal Science, Politeknik Negeri Jember, Kabupaten Jember, Indonesia

**Keywords:** Fermentation, lymph, production, protein, snail

## Abstract

**Objective:**

Protein is needed for chicken growth, but high protein consumption causes a low protein efficiency ratio and causes feed prices to be expensive. Therefore, the immunological potential of fermented snail meat hydrolysate in local chickens was studied for feed supplements in low-protein rations to reduce costs.

**Materials and Methods:**

The research used snail meat hydrolysate resulting from submerged fermentation with starter culture from rice washing water. Using hydrolysates as a fishmeal substitute to reduce the protein content of male local chicken diets. Hydrolysate is mixed into the formulated ration: P0 = 12% fish meal in feed without hydrolysate added, P1 = 8% fish meal in feed plus 5 ml hydrolysate/kg feed; P2 = 4% fish meal in feed plus 10 ml hydrolysate/kg feed; and P3 = Feed without fish meal plus 15 ml hydrolysate/kg feed. The study used completely randomized study parameters, namely the production performance of 200 chickens and the immune system (including lymphoid organs and hematological status) of 60 chickens.

**Results:**

Snail meat hydrolysate (10 ml/kg) can replace the reduction in the use of fish meal from 12% to 4% in ration formulation without reducing production performance and has no negative effect on the immune system.

**Conclusion:**

Snail meat hydrolysate has the potential to be used as an immune antibiotic to replace the use of fishmeal in the development of low-protein dietary formulations without affecting production performance or negatively affecting the chicken's immune system.

## Introduction

Sustainable poultry businesses are faced with high feed costs, especially for protein and metabolic energy requirements, which are key elements in chicken ration formulation from a cost perspective. Protein is needed in poultry feed to meet the amino acids required for growth, but high protein consumption results in low protein utilization [1], namely the chicken's ability to convert every gram of protein into body weight. Kamran et al. [2] explained that the protein content of the feed can be reduced to some extent as long as it provides optimal amino acid content for muscle growth and development. Providing poultry feed with a reduced protein content but balanced amino acid levels is advantageous, as excessive amino acids are not efficiently utilized by chickens and can hinder nitrogen excretion as uric acid. Adhering to the optimal protein composition concept, a slight decrease in protein levels, around 20–30 gm/kg, supports processing efficiency and yield; thus, decreasing the risk of foot issues and enhancing secretion quality [3,4], with no impact on chicken production efficiency or meat quality [5,6]. It has been verified that it can lower the amount of nitrogen excreted and can decrease expenses related to feed consumption [7].

Reducing protein in the ration will have an impact on the nutritional balance, which is directly involved in optimizing production performance. Balanced feed nutrition plays the most important role in supporting health and growth because it is an energy supplier so that metabolic processes can run, grow, and develop well. Utilizing low-protein feed in excessive amounts adversely affects the morphology of intestinal villi, leading to disruption [8]. Nutrient inadequacies influence the structure of the gut microbiome, intestinal function, and mucosal immune response. In chickens consuming low-protein diets, there is a reduction in the quantity of cells and the proliferation of leukocytes, ultimately affecting their productivity [9]. Reducing dietary protein intake can lead to nutritional deficiencies, exacerbating intestinal health. Moreover, it hinders the development of lymphoid organs, resulting in decreased T and B cells and a compromised immune response against antigens [10]. A decline in protein intake can alter the amino acid mix, thereby impacting the immune system through modifications in the gut microbiota [11].

Based on the description above, low-priced feed is the main concern in obtaining business efficiency, but nutritional adequacy and balance are also very important in formulating poultry rations. Hence, finding substitutes for fish meal, the costly primary protein source is essential due to the high cost. Yet, concerns arise regarding the potential adverse effects on the immune system, specifically on lymphoid organs such as the thymus, spleen, and bursa of Fabricius, critical for combating pathogens [12,13].

 The research aimed to investigate how fermented snail meat hydrolysate affects both the production performance and immune systems of local chickens. By replacing fish meal in their diet, this study sheds light on the viability of using snail meat as a protein source in free-range chicken feed. This innovative approach is crucial for formulating cost-effective rations with lower protein content without compromising chicken health or disease resistance.

## Materials and Methods

### Ethical approval

All research procedures conducted at Politeknik Negeri Jember followed the ethical standards set by the institution and Sebelas Maret University Experimental Animal Research Ethics Committee No. 741 /UMZ7. 20/ PT-O1-01/2021.

### Research procedure

The research used male free-range chickens and fermented snail meat hydrolysate made using the liquid media method (Submerged Fermentation). Fermentation of snail meat using starter culture from rice washing water. Giving hydrolysate to chickens involves blending it with a feed mix designed with reduced amounts of fish meal, according to Tables 1–3.

### Experimental design

This research is using an experimental method. 200 indigenous chickens are housed in groups, each undergoing one of four specified treatments. Treatment P0 involves a feed mix containing 12% fish meal without added hydrolysate. P1 includes 8% fish meal with 5 ml of hydrolysate per kilogram of feed. P2 comprises 4% fish meal with 10 ml of hydrolysate per kilogram of feed. Lastly, P3 consists of feed without fish meal but with 15 ml of hydrolysate per kilogram of feed. Each treatment is replicated five times. The rearing of these chickens commences at 1 day old and continues until they reach 60 days of age.

Research parameters include production performance and the immune system. Production performance parameters consist of consumption (calculating the difference between the amount of ration given and the remaining amount until 60 days old), body weight gain (the difference in final body weight minus the weight of day-old chicks divided by the length of rearing) with a total of 200 chickens. studied immune parameters encompass the hematological profile (specifically leukocytes and lymphocytes) as well as the evaluation of lymphoid organs (including the thymus, bursa of Fabricius, and the relative weight of these organs). The study utilized a total of 60 free-range chicken samples to analyze both hematological parameters and lymphoid organs, with 3 samples being taken from each replication for every treatment group

Lymphoid organs are obtained through the process of chicken slaughtering, followed by organ weighing. The next step involves calculating the organ weight as a percentage of the chicken's body weight when it reaches 60 days old. Counting the number of lymphocyte cells using the blood smear method. Place one drop of fresh blood on the slide, thin it, and spread it evenly with another slide so that a homogeneous layer of blood (blood smear) is obtained, then dry it. After drying, drip with methanol so that it covers all the blood smears, and leave for 5 min. Add 1 drop of Giemsa solution, which has been diluted in aqua distillate (1:20), and leave for 20 min. Wash with aqua distillate. Once dry, observe under a microscope. Count the number of lymphocytes at 400x magnification.

**Table 1.** Nutrient content of feed ingredients.

Feed ingredients	Nutrient content								
	ME (Kcal/kg)	CP (%)	Fat (%)	Fiber (%)	Ash (%)	Ca (%)	P (%)	Lys (%)	Met (%)
Corn	3,289.77	9.70	6.90	4.30	3.30	0.05	0.63	0.40	0.15
Rice bran	3,012.83	8.50	12.10	10.00	11.90	0.10	1.00	0.50	0.30
Soybean meal	2,998.63	41.30	4.90	5.30	8.00	0.24	0.57	2.56	0.60
Fish meal	2,742.11	52.20	6.80	2.20	20.70	5.68	3.73	3.97	1.30
Vegetable oil	8,100.00	-	-	-	-	-	-	-	-
MBM	2,375.00	43.00	10.93	2.46	6.88	9.78	4.50	2.08	0.54
Premix	-	-	-	-	-	0.60	-	3.00	3.00
CaCO_3_	-	-	-	-	-	40.00	-	-	-

**Table 2.** Treatment feed formulation for each maintenance period starter phase.

Feed ingredients	Composition (%)			
	P0	P1	P2	P3
Corn	40.68	47.6108	49.3956	56.3579
Rice bran	20.00	20.00	20.00	20.00
Soybean meal	18.32	15.3892	17.6044	13.761
Fish meal	12.00	8.00	4.00	0
Vegetable oil	2.00	2.00	2.00	2.00
MBM	5.00	5.00	5.00	5.00
Premix	1.00	1.00	1.00	0.88109
CaCO_3_	1.00	1.00	1.00	2.00
Total	100.00	100.00	100.00	100.00
Nutrient content				
Energy metabolism (Kcal/kg)	3,100.00	3,130.43	3,145.89	3,150.00
Crude protein (%)	21.63	19.00	17.00	15.00
Fat (%)	7.49	7.55	7.51	7.53
Fiber (%)	5.11	5.16	5.27	5.28
Ash (%)	8.02	7.18	6.59	5.68
Calcium (%)	1.66	1.43	1.21	1.38
Phosphor (%)	1.23	1.11	0.99	0.86
Lysin (%)	1.34	1.14	1.04	0.81
Methionine (%)	0.44	0.38	0.35	0.28

### Statistical analysis

Data underwent analysis through analysis of variance, specifically employing a completely randomized design. In cases of a significant treatment effect, a subsequent Turkey Honest Significance Difference test was conducted. The analysis was performed utilizing SPSS software.

## Results

The impact of incorporating fermented snail meat hydrolysate into chicken feed instead of fish meal has varying effects on both production performance and the immune system of free-range chickens, as outlined in Table 4. Analysis in Table 4 indicates that the consumption of feed, a key element of production performance, remains statistically unaffected (*p* > 0.05) by the reduction of fish meal and its substitution with snail meat hydrolysate during the rearing period. Similarly, the substitution of fish meal with snail meat hydrolysate at 10 ml/kg of feed from 12% to 4% does not show a notable impact (*p* > 0.05) on the chickens' final body weight gain. However, a notable difference emerges (*p* < 0.05) in cases where fish meal is entirely replaced by 15 ml/kg of fermented snail meat hydrolysate, even though in reduced quantities.

**Table 3.** Treatment feed formulation for each maintenance period finisher phase.

Feed ingredients	Composition (%)			
	P0	P1	P2	P3
Corn	40.1944	47.5264	50.3196	55.3165
Rice bran	30.00	30.00	30.00	30.00
Soybean meal	8.80558	6.08703	6.6804	4.68354
Fish meal	12.00	8.00	4.00	0.00
Vegetable oil	2.00	2.00	2.00	2.00
MBM	5.00	5.00	5.00	5.00
Premix	1.00	0.38662	1.00	1.00
CaCO_3_	1.00	1.00	1.00	2.00
Total	100.00	100.00	100.00	100.00
Nutrient content				
Energy metabolism (Kcal/kg)	3,100.00	3,150.00	3,150.00	3,144.82
Crude protein (%)	18.50	16.00	14.43	12.00
Fat (%)	8.20	8.30	8.25	8.22
Fiber (%)	5.58	5.67	5.73	5.75
Ash (%)	8.43	7.63	6.94	6.11
Calcium (%)	1.65	1.41	1.19	1.36
Phosphor (%)	1.28	1.16	1.03	0.90
Lysin (%)	1.15	0.93	0.82	0.63
Methionine (%)	0.42	0.34	0.31	0.26

The data presented in Table 4 indicates that alterations in the diet, specifically the decrease in fish meal usage by 12% and its replacement with a 15 ml/kg ration, do not have a significant impact (*p* > 0.05) on body immunity, lymphoid organs (such as the thymus and bursa of Fabricius), and hematological parameters [including leukocytes, lymphocytes, erythrocytes, hemoglobin, hematocrit, mean corpuscular volume (MCV), and mean corpuscular hemoglobin concentration (MCHC)].

**Table 4.** Production performance and immune system of local chickens.

Parameter	Treatment			
	P0	P1	P2	P3
Production performance				
Consumption (gm/chicken/day)	2,167.3^ns^	2,114.7^ns^	2,045.9^ns^	1,953.3^ns^
Body weight gain (gm/chicken/8 weeks)	744.00^a^	712.00^ab^	644.00^bc^	572.00^c^
Lymphoid organs				
Spleen	0.39 ± 0.21^ns^	0.67 ± 0.09^ns^	0.48 ± 0.18^ns^	0.49 ± 0.24^ns^
Thymus	0.20 ± 0.05^ns^	0.32 ± 0.13^ns^	0.28 ± 0.11^ns^	0.21 ± 0.05^ns^
Bursa of Fabricius	0.37 ± 0.10^ns^	0.35 ± 0.10^ns^	0.37 ± 0.16^ns^	0.36 ± 0.13^ns^
Hematology:				
Lymphocytes	92.2	91.2	90.9	91.7
Erythrocytes	2.63	2.75	2.72	2.68
Hemoglobin	15.0	17.0	16.8	15.8
Hematocrit	34.5	32.7	29.9	32.9
Leukocytes	77.47	90.22	96.36	71.90
MCV	131.2	121.7	118.4	122.8
MCHC	43.6	45.8	46.2	45.6
				

^ns^ Shows no significant difference (*p* > 0.05).Different notations indicate there is a significantly (*p* < 0.05).

## Discussion

The utilization of chicken feed did not exhibit a significant variance when switching to a low-fish meal diet, despite the reduction in protein levels in the feed. This phenomenon occurs due to the comparative energy content of the feed provided (isoprotein), which restricts the poultry's intake based on its energy content limitations. Chickens consume feed until net energy requirements are met, rather than protein requirements. Ravindran [14] reported the same thing: energy content in feed is the main determinant of poultry feed consumption. Chickens consume feed mainly to meet their energy needs. Altering the energy level of a ration influences the ration intake and necessitates adjustments to other nutritional aspects to sustain the necessary intake. As a result, the feed energy level is frequently regarded as the initial consideration in creating poultry feed formulations.

Reducing the use of fish meal in local chicken rations from 12% to feed formulated without a fish meal plus 15 ml hydrolysate/kg feed, resulting in the lowest real body weight. However, it did not significantly cause a reduction in chicken body weight by reducing the use of fish meal from 12% to 4% fish meal in the feed formulation plus 10 ml hydrolysate/kg feed. This shows that fermented snail meat hydrolysate can potentially be used as a functional liquid because it contains probiotics. Research by Suryadi et al. [15] noted that fermented snail meat hydrolysate contains 4 isolates of Lactic Acid Bacteria (LAB), which have the potential to act as probiotics and are resistant to pH values of 2.0, 2.5, 3.0, and 4.0. Probiotic microbes that live in the stomach must be tolerant to pH 3 [16] or at least pH 4 [17] and 0.2%, 0.3%, concentrations of bile salt according to Shivram and Pandav [18]. Probiotic microorganisms need to exhibit resistance to at least 0.3% bile salt concentration.

The body weight of free-range chickens fed rations without fish meal was significantly lighter than other treatments, this occurred due to the low protein content of the diet, while the LAB contained in the hydrolysate was thought to be unable to increase diet protein metabolism effectively due to the lack of secretion of endogenous proteolytic enzymes produced by probiotics. Likewise, the amino acid content contained in the hydrolysate is not sufficient for chicken growth. Zhao et al. [19] stated that a decrease in the protein content of the diet should be followed by an increase in amino acid supplementation to maintain the same growth because the balance of different amino acids in the diet will affect the utilization of nitrogen and other nutrients. Suryadi et al. [20] stated that fermented snail meat protein hydrolysate contains six types of amino acids, namely, Alanine, Glycine, Cysteine, Arginine, Lysine, and Proline. The presence of amino acid content from fermented snail meat hydrolysate can replace the lack of amino acids absorbed from dietary protein consumed due to a decrease in the use of fish meals.

### Development of lymphoid organs and hematological parameters

The findings presented in Table 4 indicate that substituting 12% of fish meal with a fish meal-free formulated feed containing an additional 15 ml of hydrolysate per kg of feed does not result in any observable decline in immune function concerning lymphoid organs and hematological metrics [21]. Chickens provided with low protein diets exhibited elevated levels of blood leukocytes, suggesting that this increase in leukocyte count was possibly influenced more by dietary protein content than hormonal factors. Malnutrition can lead to compromised immune function, marked by a diminished response to pathogens. Malnutrition, a critical state, is defined by insufficient consumption of both energy-providing macronutrients (such as carbohydrates, proteins, and fats) and essential micronutrients (including minerals and vitamins) [22].

This illustrates that hydrolysate is an immunomodulatory compound [23]. Immunomodulators refer to natural or artificial compounds that can activate or inhibit innate, adaptive, or both immune responses. The hydrolysate resulting from fermented snail meat can be used as an immunomodulatory compound because it contains amino acids and probiotics. Aiding in the synthesis of butyric acid and overall short-chain fatty acids, amino acid supplementation enhances various aspects, including growth, development, feed efficiency, and immunity levels [4,24]. The positive impacts of probiotics on the host have been confirmed through their usage in displacing harmful bacteria, enhancing the body's immune system, regulating immunity, and promoting the production of neurotransmitters [25], well-being, development progress, absorption of nutrients, and equilibrium of gut bacteria. Internal defense mechanisms [26].

The thymus, spleen, and bursa of Fabricius are organs that play an important role in cellular and humoral immunity. The thymus organs of the native chickens studied showed a tendency to increase in weight, but statistically, there was no real difference. In Table 4, it can be seen that the thymus organs of local chickens that were given a ration using fish meal reduced from 12% to feed formulated without fish meal but plus 15 ml hydrolysate/kg feed did not show any reduction in thymus weight. This is different from Quinteiro-Filho et al. [27], who state that chickens that experience a reduction in nutrients result in atrophy of the thymus organ. Feed provides nutrients for the growth and development of primary lymphoid organs, namely the bursa of Fabricius and the thymus, as well as secondary lymphoid organs, namely the spleen, mucosa-associated lymphoid tissue, and lymph nodes. Several factors influence the relative weight of the bursa of Fabricius, one of which is protein consumption. If protein consumption is low, then the growth of the bursa of Fabricius will be hampered. Another observation from our study, presented in Table 2, indicates that the reduction in protein levels resulting from the reduced utilization of fish meal does not impact the bursa of Fabricius when substituted with hydrolysate derived from fermented snail meat.

Table 4 provides a hematological picture of an increase in erythrocyte values. This indirectly aids in the assimilation of nutrients essential for the production of red blood cells. If erythrocytes increase, the amount of hemoglobin will increase. Hemoglobin is an oxygen transport tool located in erythrocytes. The hematocrit value is positively correlated with the size of erythrocytes [28]. Anemia may cause a decrease in hematocrit levels as a result of factors like diminished production of red blood cells, damage to red blood cells, and changes in their size, all without impacting the birds' overall physiological condition.

MCV measurements across all interventions within this research remained within standard ranges, indicating that the dietary requirements met by the chickens involved were satisfactory for erythrocyte production. According to Kasper et al. [29], MCV may signal folic acid deficiency when anemia is present, whereas a reduced MCV in anemia could indicate iron deficiency. The MCHC quantifies the hemoglobin content per red blood cell. The MCHC value under consideration in this research displays minimal variability [30]. The research findings regarding the MCHC value serve as an indicator of the correlation between the chicken's hemoglobin concentration and the erythrocyte count in the blood. A standard MCHC value ensures the effective functioning of hemoglobin, which is responsible for transporting oxygen from the lungs or bloodstream via erythrocytes to fulfill tissue requirements, as well as transporting carbon dioxide from the tissues to the lungs. This state significantly enhances the body's metabolic processes [28]. Hematological values such as red blood cells, hemoglobin levels, hematocrit, white blood cells, and the erythrocyte index all indicate normalcy in chickens consuming diets supplemented with fish meal. This supplementation has a favorable impact on the blood physiology of broiler chickens, suggesting their good health status.

## Conclusion

Snail meat hydrolysate shows promise as an immunobiotic, offering a viable alternative to fish meal in the creation of cost-effective, low-protein feed formulations for chickens. By incorporating 10 ml/kg of hydrolysate into the ration, the reliance on fish meal in feed production can be reduced from 12% to 4%, maintaining optimal production efficiency and supporting the chickens' immune system health.

## List of Abbreviations

MCHC, Mean corpuscular hemoglobin concentration; MCV, mean corpuscular volume; ml, milliliter; kg, kilogram.
